# Network integration meets network dynamics

**DOI:** 10.1186/1741-7007-8-48

**Published:** 2010-04-28

**Authors:** Teresa M Przytycka, Yoo-Ah Kim

**Affiliations:** 1 National Center of Biotechnology Information, NLM, NIH, 8000 Rockville Pike, Bethesda, MD 20814, USA

## Abstract

Molecular interaction networks provide a window on the workings of the cell. However, combining various types of networks into one coherent large-scale dynamic model remains a formidable challenge. A recent paper in *BMC Systems Biology *describes a promising step in this direction.

## Commentary

New high-throughput experimental techniques, complemented by recently developed computational methods, have facilitated the initial reconstructions of large-scale cellular networks. These reconstructions provide important clues about the topological organization of these networks and elucidate relationships between the topological characteristics and biological properties of the corresponding molecules. In particular, studies of protein-interaction networks have revealed complex relationships between vertex degree (number of neighbors in the network), network modularity (organization of the network into connected subnetworks), gene essentiality, gene pleiotropy, and so on. Importantly, despite considerable noise in the data, the utility of these networks goes beyond merely describing the rough landscape of biomolecular systems. They are being used increasingly to predict functionality of individual molecules in the network, membership in protein complexes, association with signaling pathways, disease-associated subnetworks, and so on (see [1] and references therein).

## Network dynamics

Experimentally and computationally derived networks, such as protein-protein interaction networks, regulatory networks or metabolic networks, provide static depictions of the dynamically changing cellular environment. Therefore, their utility for modeling cellular dynamics might not be clear. However, it is now increasingly recognized that static network topology can be used as a scaffold for studies of network dynamics. In fact, some dynamical properties can be uncovered from network topology alone, or in combination with other types of data, such as gene expression. For example, an analysis of network connectivity in terms of possible ways in which information can be propagated has been used to predict the molecules perturbed as a result of gene knockouts [1,2]. A more recent study combined protein-protein interactions, protein-DNA interactions, and phosphorylation networks with gene-expression profiles to provide a link between causative copy number variations (genetic perturbation) and molecular pathways affected in cancer [3].

Although the above-mentioned approaches provide tools for predicting which molecules are likely to be affected by a perturbation, their power to predict the changes quantitatively is extremely limited. Such quantitative predictions require knowledge of the parameters of a molecular system that goes beyond simple network connectivity. There are a number of well-established methods for quantitative modeling of dynamical systems (for a review see [4]). However, such approaches typically require knowledge of experimentally determined parameters describing the individual reactions. Consequently, these models have been developed and applied to small-scale networks only, limiting such quantitative studies to the better understood subnetworks for which such measurements can be obtained. Because such detailed data are not available on a genome-wide scale, a dynamical analysis of large-scale networks must rely on less precise methods that can estimate the behavior of the systems without knowledge of reaction parameters. For example, flux balance analysis (a mathematical approach for analyzing the flow of metabolites through a metabolic network) is often used in analyzing metabolic networks; variants of Boolean logic (a way of combining activation/inhibition signals from individual parts of a network) are frequently applied to signaling networks; and a variety of different methods have been proposed for regulatory networks (for a review see [5]).

## Modeling dynamics in large-scale heterogeneous networks

Until recently, the large-scale modeling of network dynamics has been focused on individual network types. However, within a cell, all network types are interrelated and dynamics of any individual network has an impact on the behavior of other networks. Several recent studies have begun to address the challenge of coupling large-scale dynamical models for different network types to obtain one consistent dynamical network. Such methods have been spearheaded by approaches to combine metabolic and regulatory networks (see [6-8] and references therein). For example, to obtain a combined model of metabolic and regulatory networks, Covert *et al*. [6] used flux-balance analysis to model the metabolic network component while the transcriptional regulatory network was modeled as a Boolean network. The genes in the transcriptional network were assigned Boolean (binary) values indicating whether or not a given gene is being expressed. An interactive procedure was applied to ensure that the combined model satisfies both the metabolic and the regulatory constraints. A subsequent study used mixed integer linear programming (a general optimization framework for capturing problems with both discrete and continuous variables) to couple such metabolic and regulatory models [8].

**Figure 1:**
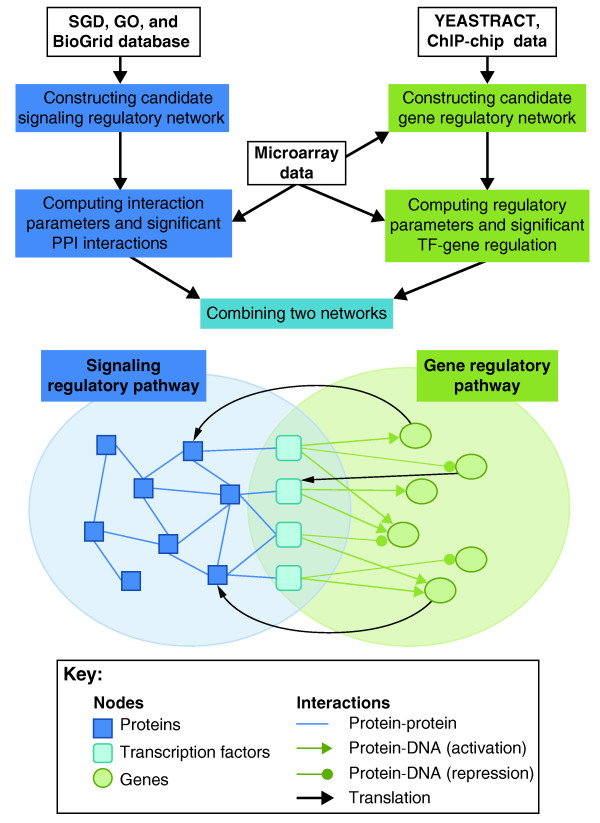
**Integrating transcriptional and signaling networks**. The figure illustrates the integration method proposed by Wang and Chen [9]. First the candidate gene regulatory network and signaling regulatory pathways are retrieved. These candidate networks are then pruned and combined, utilizing gene-expression data with time profiles to obtain an integrated and focused network under a specific condition of interest. Transcription factors serve as the interface to link the two types of networks. GO, Gene Ontology; SGD, Saccharomyces Genome Database; TF, transcription factor.

**Figure 2:**
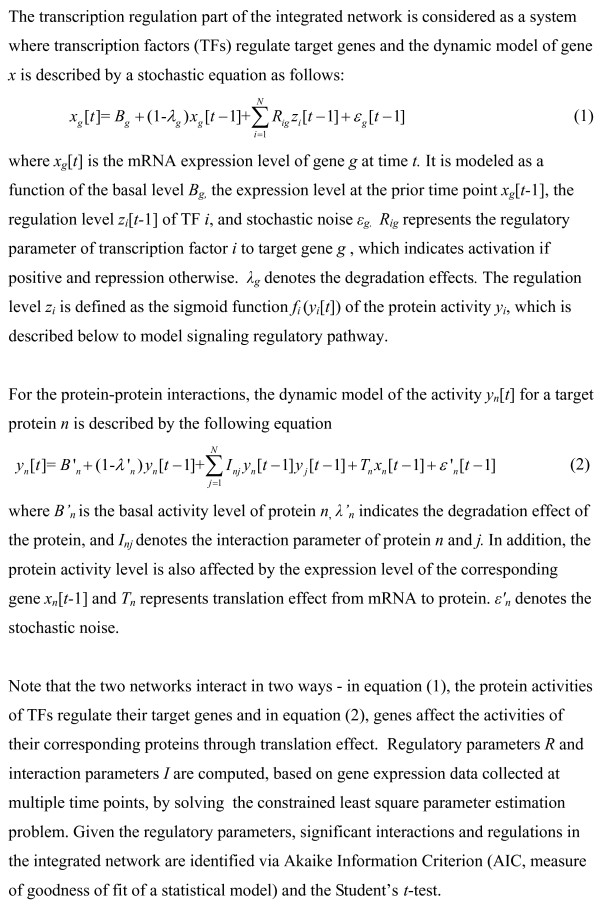
**Details of the integration method proposed by Wang and Chen **[9].

In their recent paper in *BMC Biology*, Wang and Chen [9] propose a promising approach for integrating transcription regulation and protein-protein interactions using dynamic gene-expression data. They start with candidate gene regulatory and signaling networks obtained from genome-scale data. These candidate networks are then pruned and combined, utilizing gene-expression data at multiple time points, to obtain an integrated and focused network under a specific condition of interest. The transcriptional network is modeled as a dynamical system in which the expression of a target gene (a gene subject to regulation by transcription factors included in the network) is computed as a function of regulatory impact of the corresponding transaction factors, its expression at a previous time point, and mRNA degradation rate. The modeling of a signaling/protein-interaction network takes into account, among other factors, the activities of its neighbors in the network. The interaction rate between two neighboring proteins is assumed to be proportional to the product of their concentrations. An overview of the method used by Wang and Chen [9] is depicted in Figure 1 and further details are given in Figure 2.

Wang and Chen applied their method to *Saccharomyces cerevisiae *(budding yeast) networks for three different stress responses - hyperosmotic stress, heat-shock stress and oxidative stress - and identified highly connected transcription factors and genes. Further analysis of the crosstalk between these three networks revealed the significance of some transcription factors in serving as the decision-making devices and in playing a role in rapid adaptation in the stress-response mechanism.

The authors also showed that their method can be used to predict gene-expression levels under different conditions. To do so, they first constructed the integrated network under heat-shock stress for the wild-type strain of yeast and then used the trained data to predict the expression level of the gene *HXT5 *in the *yap1 *mutant strain, which had been originally determined by Gasch *et al*. [10]. Their results suggest that various types of network models can be combined successfully to yield a predictive dynamic model of the heterogeneous system.

## Challenges and future directions

Studies of large-scale biological networks are gradually shifting from the analysis of their organizational principles and guilt-by-association predictions of the function of individual network components towards examining cell dynamics. In such studies, experimentally determined static networks are often used as scaffolds for modeling of dynamical changes in the system. Information about dynamics can be provided, for example, by measurements of gene expression at different time points or in different conditions. The methods used by Wang and Chen [9] to construct such dynamically coupled models provide important steps in this direction, but there is still much more to be done. First, the power and limitations of the new methods need to be investigated more extensively. Next, note that the combined model of protein-interaction networks and transactional network proposed by Wang and Chen [9] incorporated the impact of protein degradation, which was not included in the basic flux-balance model. In contrast, the work of Covert *et al*. [6] more accurately captured the functionality of the transcriptional network through an application of Boolean logic. Obviously, both approaches are important and should be considered in future models. Finally, an approach that combines all three networks is still lacking. Indeed, much has to be done before genome-scale models will be able to approximate cell dynamics with a precision close to what is expected from differential equation methods. But keeping in mind that a great deal was learned from the static models alone, we expect that even simple genome-wide scale dynamical models will bring further interesting discoveries.

## Authors' information

TMP is an Investigator in the National Center for Biotechnology Information (NCBI), National Library of Medicine (NLM), National Institutes of Health (NIH), where she heads a research group focused on algorithmic and graph theory methods in computational and systems biology. YK is a research fellow in NCBI\NLM\NIH where she works on developing computational methods for the genome-wide, systems-level analysis of cell biology.
